# Ancient origin and maternal inheritance of blue cuckoo eggs

**DOI:** 10.1038/ncomms10272

**Published:** 2016-01-12

**Authors:** Frode Fossøy, Michael D Sorenson, Wei Liang, Torbjørn Ekrem, Arne Moksnes, Anders P Møller, Jarkko Rutila, Eivin Røskaft, Fugo Takasu, Canchao Yang, Bård G Stokke

**Affiliations:** 1 Department of Biology, Norwegian University of Science and Technology (NTNU), Høgskoleringen 5, NO-7491 Trondheim, Norway; 2 Department of Biology, Boston University, Boston, Massachusetts 02215, USA; 3 Ministry of Education, College of Life Sciences, Hainan Normal University, Haikou 571158, China; 4 Department of Natural History, NTNU University Museum, Norwegian University of Science and Technology, NO-7491 Trondheim, Norway; 5 Ecologie, Systématique et Evolution, UMR 8079 CNRS, F-91405, Orsay Cedex; Université Paris-Sud 11, F-91405, Orsay Cedex, AgroParisTech, F-91405 Orsay, France; 6 Department of Biology, University of Eastern Finland, FI-80101 Joensuu, Finland; 7 Department of Information and Computer Sciences, Nara Women's University, Kita-Uoya, Nishimachi, Nara 630-8506, Japan

## Abstract

Maternal inheritance via the female-specific W chromosome was long ago proposed as a potential solution to the evolutionary enigma of co-existing host-specific races (or ‘gentes') in avian brood parasites. Here we report the first unambiguous evidence for maternal inheritance of egg colouration in the brood-parasitic common cuckoo *Cuculus canorus*. Females laying blue eggs belong to an ancient (∼2.6 Myr) maternal lineage, as evidenced by both mitochondrial and W-linked DNA, but are indistinguishable at nuclear DNA from other common cuckoos. Hence, cuckoo host races with blue eggs are distinguished only by maternally inherited components of the genome, which maintain host-specific adaptation despite interbreeding among males and females reared by different hosts. A mitochondrial phylogeny suggests that blue eggs originated in Asia and then expanded westwards as female cuckoos laying blue eggs interbred with the existing European population, introducing an adaptive trait that expanded the range of potential hosts.

## Abstract (web summary)

The common cuckoo lays its eggs in nests of a variety of species and their eggs mimic the ones of their hosts. Here, the authors show that blue egg colouration in the common cuckoo is maternally inherited, originated in Asia and then expanded to Europe.

## Graphical abstract

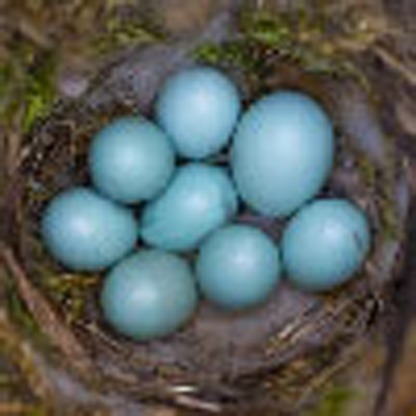

Adaptations and counter-adaptations in the context of co-evolutionary arms races between parasites and their hosts can drive rapid phenotypic change^1^. Generalist parasites may express distinct host-specific adaptations that match particular host species, but the genetic basis of these traits is poorly understood^1^. The common cuckoo *Cuculus canorus* is a generalist obligate parasite, exploiting a variety of passerine species across Eurasia, and it has evolved numerous host-specific races (termed ‘gentes'), each having eggs with colour and pattern mimicking the eggs of their respective host species^2^,^3^. In Europe, 15–20 host-specific egg morphs are recognized, although cuckoo eggs have been found in the nests of more than 100 different host species^2^. The genetic architecture of egg colouration and the evolutionary maintenance of host-specific races in the cuckoo remain largely unknown, but female heterogamy in birds (females have one Z and one W chromosome while males have two Z chromosomes) has led to the hypothesis that the genes controlling egg colour are located on the female-specific W chromosome, thus resulting in maternal inheritance of egg traits and allowing the evolution of host-specific gentes without assortative mating^4^,^5^,^6^. Others have argued that maternal inheritance is unlikely given that egg colour is inherited from both parents in other birds and that the small and uniform W chromosome is unlikely to harbour sufficient genetic variation to explain the vast diversity of egg colouration in cuckoos^7^. The first specific gene for egg colour in any bird species was recently discovered in the domestic chicken *Gallus gallus*, in which blue egg colour is associated with an autosomal, endogenous retroviral insertion, resulting in a dominant Mendelian trait^8^.

We tested these alternative hypotheses with an expanded geographic sampling of common cuckoos (Fig. 1) and a particular focus on immaculate blue eggs, which are perhaps the most distinctive of all common cuckoo eggs (Fig. 2a)^9^. In Europe, blue cuckoo eggs are relatively rare, comprising only 3% of almost 12 000 eggs in museum collections^9^. Blue cuckoo eggs in Europe are mainly found in the nests of common redstarts *Phoenicurus phoenicurus*, where they are excellent mimics of the host's pale blue eggs^9^. In Asia, the common cuckoo subspecies *C. c. bakeri* lays blue eggs in the nest of several host species, and they have evolved polymorphic eggs with different intensities of blue matching a similar egg polymorphism in their parrotbill *Paradoxornis spp.* hosts^10^,^11^,^12^. In the following analyses, ‘blue' eggs include all immaculate cuckoo eggs with varying intensities of blue colouration.

Here we find that all female cuckoos laying blue eggs in Europe belong to a highly divergent maternal lineage shared with the Asian subspecies, but are indistinguishable at nuclear loci from other common cuckoos. The blue egg colour is therefore maternally inherited, supporting the hypothesis that the genes controlling egg colour are located on the female-specific W chromosome.

## Results

### Population genetic analyses

**Figure 1:**
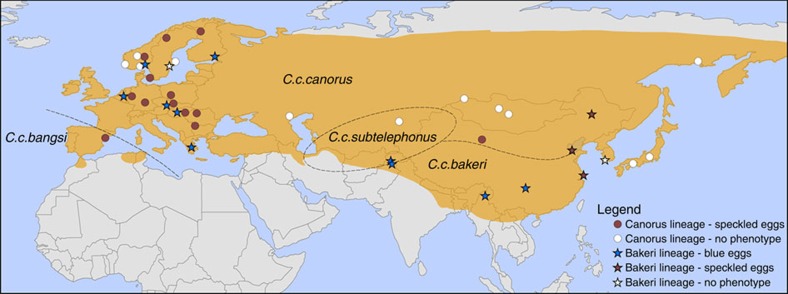
DNA sampling. Breeding distribution (shaded) of the four common cuckoo *Cuculus canorus* subspecies^44^ along with DNA sampling localities coded to illustrate the geographic distribution of two divergent mitochondrial lineages and their corresponding egg colours. All cuckoos laying blue eggs belong to the same ancient mitochondrial lineage (labelled above as ‘*bakeri*' but also including *C. c. canorus* blue eggs) but are indistinguishable from other common cuckoos at nuclear loci. The blue egg colour is therefore maternally inherited. A recent split within *C. c. bakeri* is associated with a loss of blue egg colour in eastern Asia. The map is constructed in ArcMap 10.1.

**Figure 2:**
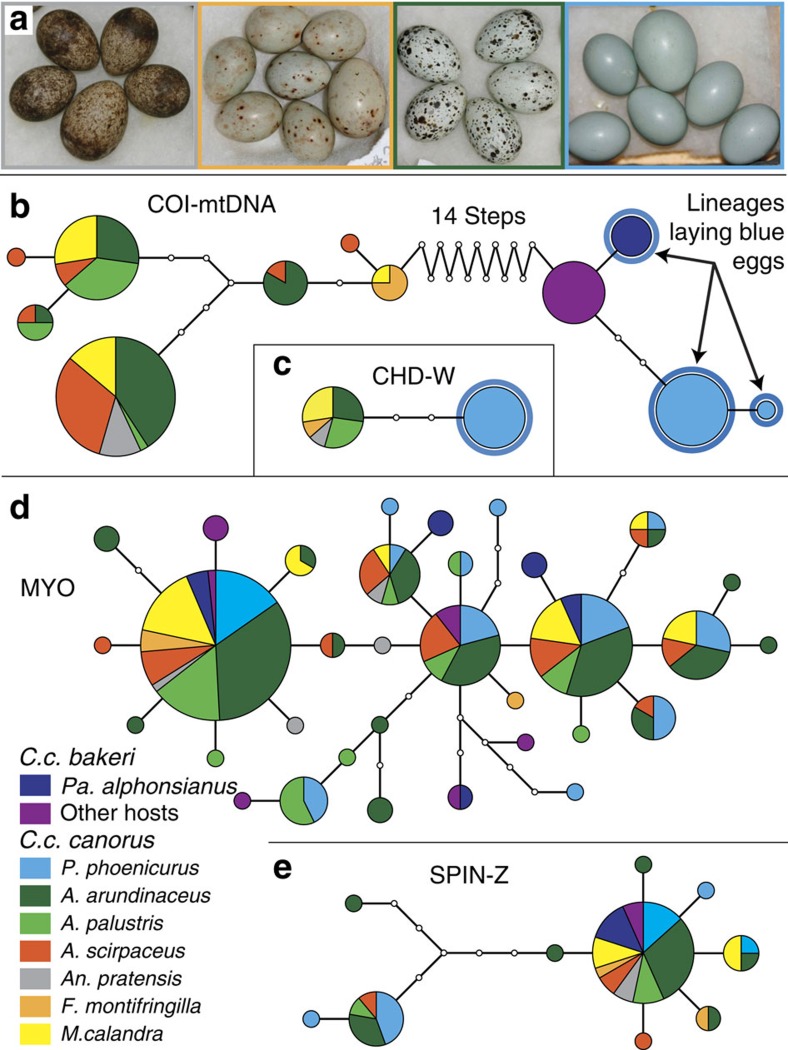
Egg phenotypes and haplotype networks. (**a**) Examples illustrating host-specific egg mimicry and the tremendous variation in egg colour among common cuckoo host races. From left: meadow pipit *Antus pratensis*, brambling *Fringilla montifringilla*, great reed warbler *Acrocephalus arundinaceus* and common redstart *Phoenicurus phoenicurus* (in each clutch, the cuckoo egg is slightly larger). All photos by B.G.S. Haplotype networks for (**b**) COI—mitochondrial DNA, (**c**) CHD-W—nuclear W chromosome, (**d**) MYO—nuclear autosomal (2N) and (**e**) SPIN—nuclear Z chromosome, all sequenced in common cuckoo host races from Europe and Asia. Each coloured circle represents a unique haplotype, the relative frequency of which in our sample is proportional to area. Samples from females of different host races are colour-coded as indicated. Each line segment represents a single nucleotide difference, with small open circles indicating intermediate haplotypes not found in our sample. Sample size for the autosomal MYO gene is larger because each sample yields two alleles.

**Figure 3:**
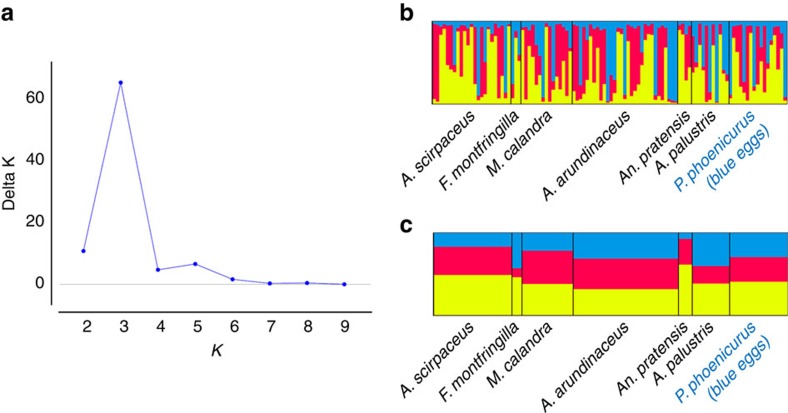
STRUCTURE analysis. (**a**) The Evanno method implemented in STRUCTURE HARVESTER^35^ suggests that the most likely number of common cuckoo *Cuculus canorus* genetic groups in Europe is *K*=3 based on 13 microsatellite markers and 104 individuals. (**b**) Individual and (**c**) population Q matrices for the three genetic groups show little evidence that this putative population structure is related to either host race or the divergent mitochondrial lineages.

In Europe, 197 common cuckoo offsprings originating from 7 host races were assigned to 104 inferred mothers using COLONY^13^ (Supplementary Table 1, see Methods for details). We randomly selected one offspring representing each inferred mother for further analyses. Population genetic analyses of pairwise genetic divergence revealed that cuckoos parasitizing redstarts in Finland, and laying immaculate blue eggs, were highly divergent from the other European host races in both their mitochondrial DNA (mtDNA; COI—*F*_st_ range: 0.898–0.995) and W chromosome DNA, but not at autosomal or Z-linked loci (MYO and SPIN—*F*_st_ range: 0.013–0.216, Fig. 2, Supplementary Table 2). Likewise, a STRUCTURE^14^,^15^,^16^ analysis based on 13 nuclear microsatellite markers suggested *K*=3 as the most likely number of genetically distinct groups within our European samples, but this putative structuring was unrelated to the two mitochondrial lineages or host race identity (Fig. 3). Expanding the analysis to include samples from Asian and African cuckoos (Supplementary Tables 3 and 4) revealed that all individuals of the *C. c. bakeri* subspecies are closely related to the divergent mitochondrial lineage associated with blue eggs in Finland, suggesting that the Finnish redstart cuckoos share a recent matrilineal ancestor with the *C. c. bakeri* subspecies (Figs 1 and 2, Supplementary Figs 1 and 2). The genetic divergence within this lineage was relatively small (0.14–0.69%) compared with that between the two major lineages (2.15–3.86%), with the latter divergence being comparable to that between species (Table 1, Supplementary Table 5).

### Analyses of museum eggs

Genotyping of cuckoo eggs stored in European museum collections, including multiple host races from a large geographical distribution, corroborated a strict association between egg colour and mtDNA with all blue eggs being part of the divergent ‘*bakeri*' lineage (including the Finnish redstart cuckoos) and all non-blue eggs being part of the more common ‘*canorus*' lineage (Fig. 1, Supplementary Tables 6 and 7). In particular, blue (*n*=24) and non-blue (*n*=9) cuckoo eggs collected from a number of different host species in a single sympatric population were strictly associated with the two different mitochondrial lineages (Supplementary Table 6). Hence, blue egg colouration must be maternally inherited.

### Phylogenetic analyses

**Figure 4:**
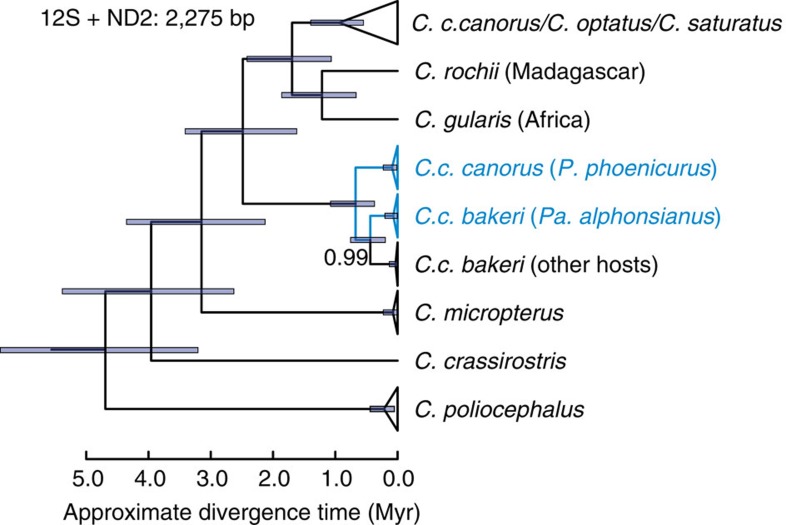
Phylogenetic relationships and dating. Mitochondrial phylogeny and approximate divergence times for the common cuckoo *Cuculus canorus* and closely related species. The analysis is based on a comprehensive cuculiform data set representing 119 of 141 recognized cuckoo species^21^ augmented with additional data for representative samples from this study (see Supplementary Fig. 2 for the complete phylogeny). Blue colour indicates common cuckoo lineages laying immaculate blue eggs. All nodes have posterior probabilities of 1, except as indicated. Approximate divergence times are shown with 95% highest posterior density intervals. See Supplementary Information for details on samples and calibration.

A comprehensive mitochondrial phylogeny of 119 Cuculiform species suggests that the divergent lineage associated with blue egg colour split from the other *Cuculus* cuckoos roughly 2.6±0.8 Myr ago (Fig. 4, Supplementary Fig. 2). This ancient split preceded the diversification of several well-established species in the genus (including *C. gularis* in Africa and *C. rochii* in Madagascar), but autosomal and Z-linked markers bear no evidence that would support splitting the common cuckoo into two species (Supplementary Fig. 1). Within the ‘*bakeri*' mitochondrial lineage, the European cuckoos laying blue eggs separated from their Asian relatives an estimated 0.67±0.40 Myr ago (Fig. 4). The phylogeny also reveals a more recent split (0.44±0.25 Myr ago) within *C. c. bakeri*, which appears to be associated with a host shift and reversal in egg colouration (Fig. 4).

## Discussion

Our analyses of mtDNA and W-linked DNA show that all common cuckoo females laying immaculate blue eggs belong to an ancient maternal lineage. In marked contrast, autosomal and Z-linked genetic markers fail to distinguish European cuckoos belonging to the two mitochondrial lineages (Fig. 2). Hence, males evidently mate across the two female lineages, resulting in extensive nuclear gene flow and preventing speciation^7^,^17^. Blue egg colouration is therefore maternally inherited because any significant paternal/autosomal contribution to the phenotype would have quickly disrupted the perfect association between mitochondrial lineage and egg colour. This provides strong evidence for the hypothesis that the gene(s) responsible for blue colouration as well as the lack of additional markings (maculation) is (are) located on the female-specific W-chromosome, assuming that pleiotropic effects of one or more mitochondrial genes on egg phenotype is an unlikely mechanism. The plausibility of selection generating adaptation through changes in one or more W-linked genes is strengthened by a recent study showing that increased female-specific selection in chickens during domestication has resulted in increased expression of W-linked genes^18^. Female heterogamy (ZW) is also found in butterflies, and an analogous situation is observed in the tiger swallowtail *Papilio glaucus*, in which polymorphic female-specific Batesian mimicry is linked to the W chromosome^19^.

As noted above, disruptive selection in the context of a co-evolutionary arms-race has apparently resulted in colour polymorphism in both the common cuckoo and its parrotbill hosts in Asia, with both species laying immaculate eggs varying from blue to almost white^12^. Immaculate cuckoo eggs from European collections also vary in the intensity of blue colouration, with both dark blue and pale blue eggs being observed in redstart host nests in central Europe^20^. This variation in colour was also noted by the original collectors^20^ and is therefore unlikely due to differential fading over time. Thus, the novel ‘blue' W-linked gene hypothesized above may code for immaculate eggs with varying degrees of blue pigmentation depending on interactions with one or more autosomal loci and/or environmental factors.

Placing the two divergent common cuckoo mitochondrial lineages in a broader phylogenetic context^21^ suggests a divergence time of 2.6±0.8 Myr, well before the diversification of several related cuckoo species in Asia and the Southern Hemisphere (Fig. 4). This timeframe roughly coincides with the onset of the Pleistocene glacial periods as well as the end of the major tectonic uplift of the Himalayas^22^,^23^, suggesting that geographic isolation may have played an important role in the initial divergence of the two lineages. Host–parasite coevolution, however, is also critical for explaining the unusual co-occurrence of highly divergent mtDNA lineages in the contemporary European cuckoo population. Blue eggs appear to have originated in the ancestral maternal lineage from which both Asian *C. c. bakeri* and European *C. c. canorus* with blue eggs are descended. A likely scenario is that blue eggs evolved in Asia, with strong selection imposed by host egg recognition and rejection favouring a novel W-linked mutation in an ancestral cuckoo population associated with hosts having blue eggs. Subsequently, although perhaps not recently (note the estimated 0.67±0.40 Myr divergence of the European blue egg lineage from *C. c. bakeri* in Fig. 4), this lineage expanded westwards, introgressing into existing cuckoo populations in Europe and introducing an adaptive trait that facilitated the exploitation of new hosts. Several species of redstarts laying blue eggs currently breed throughout the Middle East and Russia and these may have facilitated the westward spread of blue eggs into Europe. This adaptive introgression on secondary contact left intact the non-recombining mitochondria and W chromosome lineages that we hypothesize were inherited from Asian ancestors but thoroughly mixed the nuclear genome, resulting in the genetic patterns observed today^24^. Interestingly, a more recent mitochondrial split within the Asian *C. c. bakeri* subspecies separates cuckoos with immaculate blue eggs from cuckoos that parasitize other host species and lay speckled, non-blue eggs (Fig. 4), a pattern also consistent with maternal inheritance and suggesting a recent loss of the blue egg trait associated with a host shift. Whether other egg colours and spotting patterns in European common cuckoos are maternally inherited remains an open question.

A previous study found evidence of assortative mating among three sympatrically breeding host races^7^, consistent with the theoretical possibility of bi-parentally inherited host-specific adaptations evolving in a divergence-with-gene-flow context. On the other hand, subtle differentiation in mtDNA haplotype frequencies has been reported for several host races^6^,^7^, suggesting fidelity of female lineages to hosts over evolutionarily significant timescales. However, no other gens or egg colour phenotype is known to be monophyletic for mtDNA. If inheritance is maternal in other gentes, it would imply the independent evolution of egg mimicry in each female lineage independently colonizing a particular host and, in effect, multiple origins of each host race in space and time^6^,^7^. A similar genetic pattern has been reported in the brood-parasitic greater honeyguide *Indicator indicator*, in which two highly divergent female lineages parasitize ground- or tree-nesting hosts, respectively, but in which there is also significant host-specific variation in egg traits within the tree-nesting lineage despite little or no mitochondrial differentiation^25^.

Extreme intraspecific polymorphism in egg colouration in the hosts of some brood parasites is thought to be an effective anti-parasite adaptation maintained by frequency-dependent selection^12^,^26^. However, there is no expectation of maternal inheritance of egg colouration in hosts because recombination should be advantageous for generating novelty, which increases the host's ability to detect parasitic eggs. In the village weaver *Ploceus cucullatus*, a species with highly variable eggs and a common host of the diederik cuckoo *Chrysococcyx caprius* in Africa, the available evidence suggests that at least two autosomal loci control eggshell colouration^27^.

While the specific gene(s) and molecular mechanisms producing blue eggs remain unknown, our data provide the first unambiguous evidence for the long-standing hypothesis that egg colouration in brood-parasitic cuckoos is maternally inherited. In addition, female cuckoos laying blue eggs in Europe are the matrilineal descendants of an ancient lineage that likely evolved in Asia, providing a remarkable example of the geographic expansion and selective introgression of an adaptive trait into an existing population.

## Methods

### Sample collection

Genetic samples from 205 European common cuckoos were collected from 7 different host races originating from 8 countries (Fig. 1, Supplementary Table 1). Genetic samples from several related cuckoo species were collected in Asia and Africa (Supplementary Table 3). DNA was collected either through blood sampling of cuckoo nestlings or sampling embryonic tissue from ejected/unhatched eggs laid in the nests of different host species. We also collected DNA samples from eggs in museum collections by slightly increasing the size of the ‘blow hole' using a handheld drill and extracting DNA from the resulting finely ground eggshell powder (Supplementary Tables 6 and 7). Samples for genetic analyses were preserved in 96% ethanol for subsequent analyses.

### Genetic data collection

All samples from Europe were processed in the genetics lab at the Norwegian University of Science and Technology, Trondheim, Norway. DNA was extracted from blood or tissue samples using the E.Z.N.A. blood DNA kit (Omega Bio-Tek Inc, Norcross, USA). Multiple mitochondrial and nuclear genetic loci were sequenced (Supplementary Table 8) and 13 microsatellite loci (Supplementary Table 9) were amplified and genotyped. All loci were amplified by PCR on a GeneAmp PCR System 9700 (Applied Biosystems, Foster City, USA) and run on a 3130XL Genetic Analyser (Applied Biosystems). Microsatellite data were scored in GENEMAPPER v.3.7 (Applied Biosystems) and sequence data were assembled and manually checked in GENEIOUS v.4.7.6 (Biomatters, Auckland, New Zealand). To ensure consistency, one person (FF) scored all of the microsatellite genotypes. The sequencing of DNA samples originating from Asia and from museum eggshells was outsourced to Ecogenics GmbH, Zürich-Schlieren, Switzerland. Mitochondrial 12S and ND2 gene sequences for selected European samples were also generated by Ecogenics GmbH. For amplification of DNA extracted from eggshell samples, we designed a new pair of primers using Primer3 (ref. 28) to amplify a short, 159-bp region of COI. The primer pair was designed to amplify a region distinguishing the two divergent mitochondrial lineages. We also designed new primers to amplify a portion of the Z-linked Spindlin gene in African and Madagascar cuckoos. Likewise, new primers were designed to amplify a 443-bp region of the W-linked CHD-W gene. W-specificity of these primers was corroborated by testing the primer pair on six different male samples, all of which failed to produce a product. See Supplementary Table 8 for primer sequences.

### Population genetic analyses

Many cuckoo nestlings were collected within the same local area and could be either full- or half-siblings. Thus, to avoid pseudo-replication of the cuckoo lineages associated with a given host, we used COLONY^13^ to identify full and half siblings. In contrast to similar software that only considers pairwise comparisons, COLONY utilizes a full-pedigree likelihood approach, which considers the likelihood of the entire pedigree structure and allows for the simultaneous inference of parentage and sib-ships. Moreover, COLONY allows the user to add information on known relationships among the offsprings to increase the probability of correctly assigning individuals to sib-ships. We therefore included information on geographic locality, mitochondrial haplotype and egg appearance to inform the inference of sibling relationships. Offsprings sampled from distant localities are not likely to share either the same father or mother, whereas offsprings with different mitochondrial haplotypes are highly unlikely to share the same mother. Finally, two offsprings having identical mitochondrial haplotypes, but originating from eggs of different appearance are also unlikely to share the same mother. The appearance of eggs from a given cuckoo female is highly repeatable and can be used to assign eggs to individual females, although different females may produce similar eggs^29^. Some offsprings were assigned as full- or half-siblings by COLONY, but showed a low probability of sib-ship in the analysis. Therefore, we conservatively removed one of the individuals assigned to each pair of putative siblings with a probability lower than 0.95. Of the 205 offsprings analysed for 13 microsatellite loci (mean=12.9, range=10–13 loci genotyped per sample), 8 offsprings were therefore excluded from further analyses. This left us with 197 offsprings assigned to 104 different mothers for the population genetic analyses. For the sequence-based genetic markers, we utilized subsets of the main data set, including only one offspring per female for statistical analyses. A few siblings were included in the haplotype network for the myoglobin gene, as full siblings often have different genotypes at autosomal loci.

To test for departures from Hardy–Weinberg equilibrium and linkage equilibrium among the microsatellite markers, we randomly selected one offspring per inferred female parent (*N*=104). We used exact tests as implemented in the software ARLEQUIN v. 3.5 (ref. 30) to test for possible departures from Hardy–Weinberg equilibrium for each marker across all host races (100,000 dememorization steps and 1,000,000 Markov chain steps). Although three markers showed significant deviation from Hardy–Weinberg equilibrium (Supplementary Table 9), none of them remained significant after Bonferroni correction and none of them showed any consistency across host races. Hence, we considered all markers to be in Hardy–Weinberg equilibrium. We used the software FSTAT v. 2.9.3.2 (ref. 31) and the genotypic disequilibrium function to test for linkage among loci. We found no evidence of genotypic disequilibrium between any pair of microsatellite markers^7^ and hence considered all markers to be unlinked.

We used a hierarchical *F*_st_ analysis as implemented in the package HIERFSTAT^32^ in the statistical software R^33^ to compute microsatellite pairwise genetic differentiation among host races. This approach allows for the inclusion of all offsprings while controlling statistically for shared parentage. In a hierarchical *F*_st_ analysis, each level of population structure is tested independently of the effect of lower levels in the hierarchy. HIERFSTAT uses a likelihood ratio G-statistic and randomizes the diploid genotypes rather than the alleles^32^, which is more appropriate for diploid organisms^34^. We used 10,000 permutations for testing the level of significance. One offspring representing each of the 104 inferred mothers were included in a STRUCTURE^14^,^15^,^16^ analysis of microsatellite loci to detect the most likely number of genetically distinct groups. The analysis was based on the Admixture model with burn-in set to 100,000 followed by 500,000 MCMC replicates. The output from STRUCTURE was analysed in STRUCTURE HARVESTER^35^, which implements the Evanno method to infer the most likely number of differentiated populations, and the results were visualized using DISTRUCT 1.1 (ref. 36).

Pairwise genetic differentiation among host races and subspecies at the sequenced loci was quantified as *Φ*_ST_^37^, a sequence-based analogue of *F*_st_, using ARLEQUIN and tested for significance using 1,000 permutations of the data. Alleles at MYO were inferred using PHASE v. 2.1.1 (refs 38, 39). The SPIN data were collected primarily from females, which are heterogametic and thus have only one copy of this Z-linked gene. Haplotype networks were constructed based on results from TCS^40^.

### Phylogenetic analyses

We compiled partial sequences of the mitochondrial COI gene for 139 *Cuculus* cuckoos (see above for details) and, to serve as an outgroup, three species of *Hierococcyx* cuckoos (*n*=2 samples per species); *Hierococcyx* is the sister genus to *Cuculus*^21^. We augmented this data set with 16 additional *Cuculus* COI sequences and all other available cuculiform COI sequences from GenBank (www.ncbi.nlm.nih.gov/genbank) and Barcode of Life Data Systems (www.boldsystems.org). Given inconclusive resolution of critical ingroup relationships in the COI tree, we generated mitochondrial 12S ribosomal RNA and ND2 gene sequences for representative samples of each *Cuculus* mitochondrial lineage and combined these data with the comprehensive cuculiform data set from Sorenson and Payne^21^. The latter data set provides a more comprehensive sampling of species-level *Cuculus* lineages and, with three times as much sequence data, more robust estimates of relationships and relative divergence times. For both the COI and 12S/ND2 data sets, we completed analyses both with a broad sampling of Cuculiform taxa and with *Hierococcyx* only as the outgroup to *Cuculus*. Results for all analyses, including phylogenetic relationships and relative divergence times were broadly consistent except for the placement and divergence time of *C. micropterus*, which differed between the COI and 12S/ND2 data sets. For each data set, we used jModelTest2 (ref. 41) to determine the best-fit model of nucleotide substitution based on the Bayesian Information Criterion. For the 12S/ND2 analyses, we partitioned the data set into ND2 codon positions (1st, 2nd, 3rd) and RNA regions and fitted separate substitution models to each partition, but kept tree topology and branch lengths linked across partitions. Phylogenetic analyses were completed in BEAST v. 1.7.2 (ref. 42) employing a relaxed molecular clock model with an uncorrelated lognormal distribution of evolutionary rates among lineages^43^. Each analysis was run for 50M generations, sufficient to yield effective sample size (ESS) values of ⩾200 for all parameters. For each data set, results based on different coalescent priors were nearly identical. Here we only show results for the birth–death speciation prior.

As with other birds, there is a paucity of unambiguous fossil calibration points within cuckoos^44^, and the use of external calibrations is complicated by the generally poor resolution of relationships among neoavian orders^45^. Thus, to provide an approximate timescale for the divergence of *Cuculus* mitochondrial lineages, we used the midpoint of the range of previous molecular estimates for the basal split between the New World cuckoos (comprising Crotophaginae and Neomorphinae) and the predominantly Old World clade comprising all other cuckoos^21^. Point estimates for the basal divergence of extant cuckoos range from ∼47 to 79.5 Myr (Supplementary Table 10), a range that reflects the continuing debate over whether extant avian orders originated before or after the K–T boundary 46,47. We used the midpoint of this range as the estimate of the age of the cuculiform crown group, assuming a normally distributed prior with a s.d. of 8 Myr (63±8 Myr). As an additional, simple metric of relative divergence times, we also calculated average pairwise sequence divergence between selected *Cuculus* lineages using substitution models and parameter values as determined by jModelTest (Table 1).

## Additional information

**Accession codes:** DNA sequences generated in this study have been deposited in the European Nucleotide Archive (ENA) under accession codes LN734262 to LN734648 and LN876762 to LN876780.

**How to cite this article:** Fossøy, F. *et al*. Ancient origin and maternal inheritance of blue cuckoo eggs. *Nat. Commun.* 7:10272 doi: 10.1038/ncomms10272 (2016).

## Supplementary Material

Supplementary Information Supplementary Figures 1-2, Supplementary Tables 1-10 and Supplementary References

## Figures and Tables

**Table 1:** Pairwise genetic divergence between selected *Cuculus* lineages for three mitochondrial genes.

**Comparison**	**COI**	**ND2**	**12S**
*C. c. bakeri (Pa. alphonsianus)*—*C. c. bakeri (other hosts)*	0.14%	0.64%	0.22%
*C. canorus (P. phoenicurus)*—*C. c. bakeri*	0.52%	0.69%	0.71%
*C. canorus (P. phoenicurus)/bakeri*—*C. canorus/optatus/saturatus*	3.16%	3.86%	2.15%
*C. micropterus*—*C. canorus(P. phoenicurus)/bakeri*	2.91%	4.99%	3.43%
*C. micropterus*—*C. canorus/optatus/saturatus*	3.33%	4.27%	2.16%
*C. poliocephalus*—*C. ‘canorus'/micropterus*	5.11%	8.21%	5.09%
Substitution model	TrN+I	TrN+G	HKY+I

Common cuckoos *Cuculus canorus* parasitizing common redstarts *Phoenicurus phoenicurus* in Finland and Ashy-throated parrotbills *Paradoxornis alphonsianus* in China lay immaculate blue eggs.
