# Supplementary material for: Ancient origin and maternal inheritance of blue cuckoo eggs
Source: Nat Commun. 2016 Jan 12;7:10272. doi: 10.1038/ncomms10272 (PMC4729921; doi:10.1038/ncomms10272)
Supplement: Supplementary Information — Supplementary Figures 1-2, Supplementary Tables 1-10 and Supplementary References [file ncomms10272-s1.pdf]

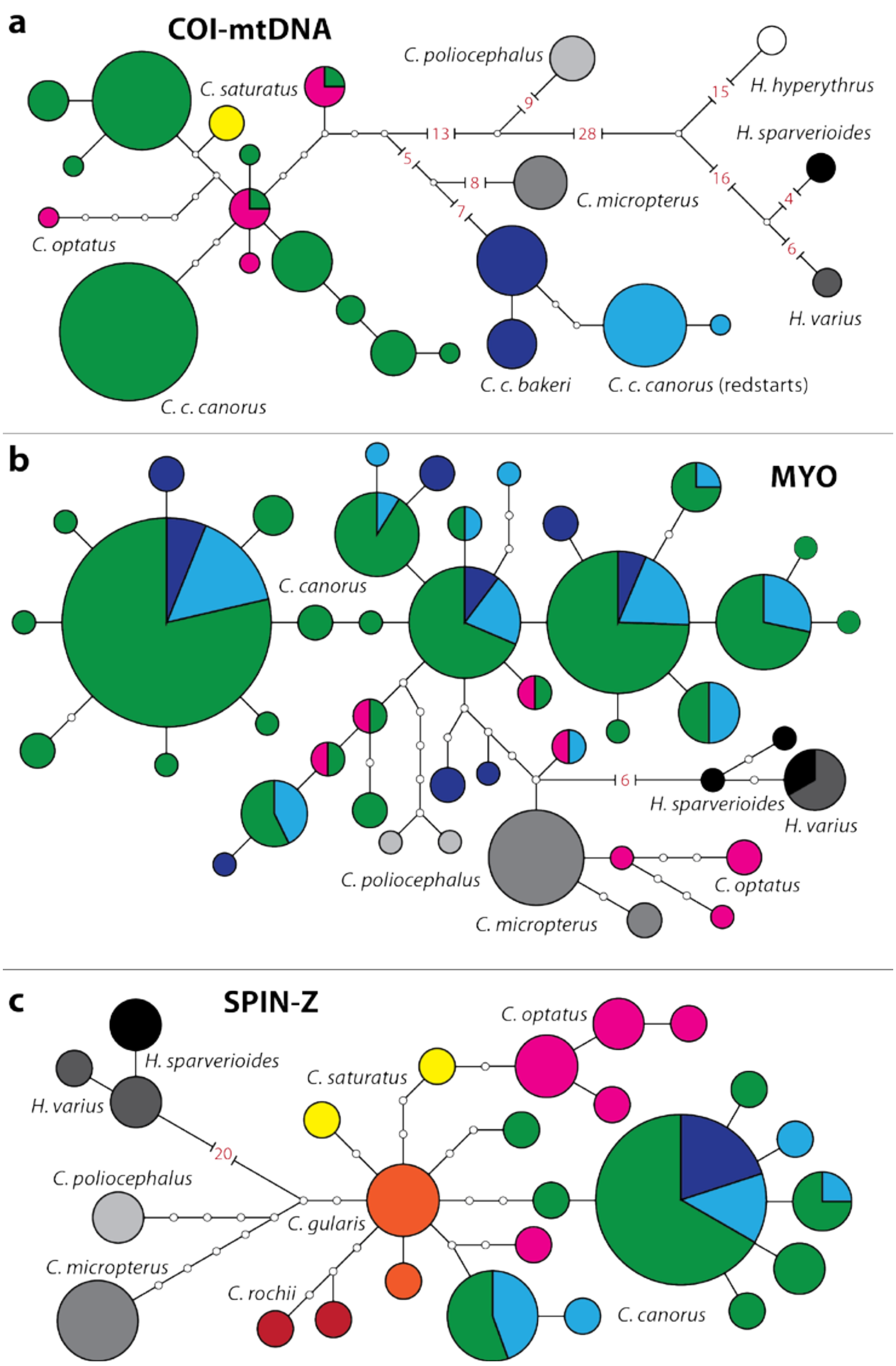

Supplementary Figure 1 | Extended haplotype networks for the common cuckoo *Cuculus canorus* in relation to other *Cuculus* and *Hierococcyx* species. (**a**) COI – mitochondrial DNA, (**b**) MYO – nuclear autosomal DNA (2N) and (**c**) SPIN – nuclear Z chromosome DNA. Each coloured circle represents a unique haplotype, the relative frequency of which in our sample is proportional to area. Species are labelled and colour-coded with Asian *C. c. bakeri* in dark blue, *C. c. canorus* females parasitizing redstarts *Phoenicurus phoenicurus* in light blue (both groups having blue eggs) and remaining *C. c. canorus* in green (having speckled eggs). Each line segment represents a single nucleotide difference, with small open circles indicating intermediate haplotypes not found in our sample. Red numbers denote number of nucleotide differences between two haplotypes. The haplotype networks illustrate the close phylogenetic relationship between European blue-egg redstart cuckoos and *C. c. bakeri* for mitochondrial DNA and the contrasting lack of a similar pattern for the two nuclear markers.

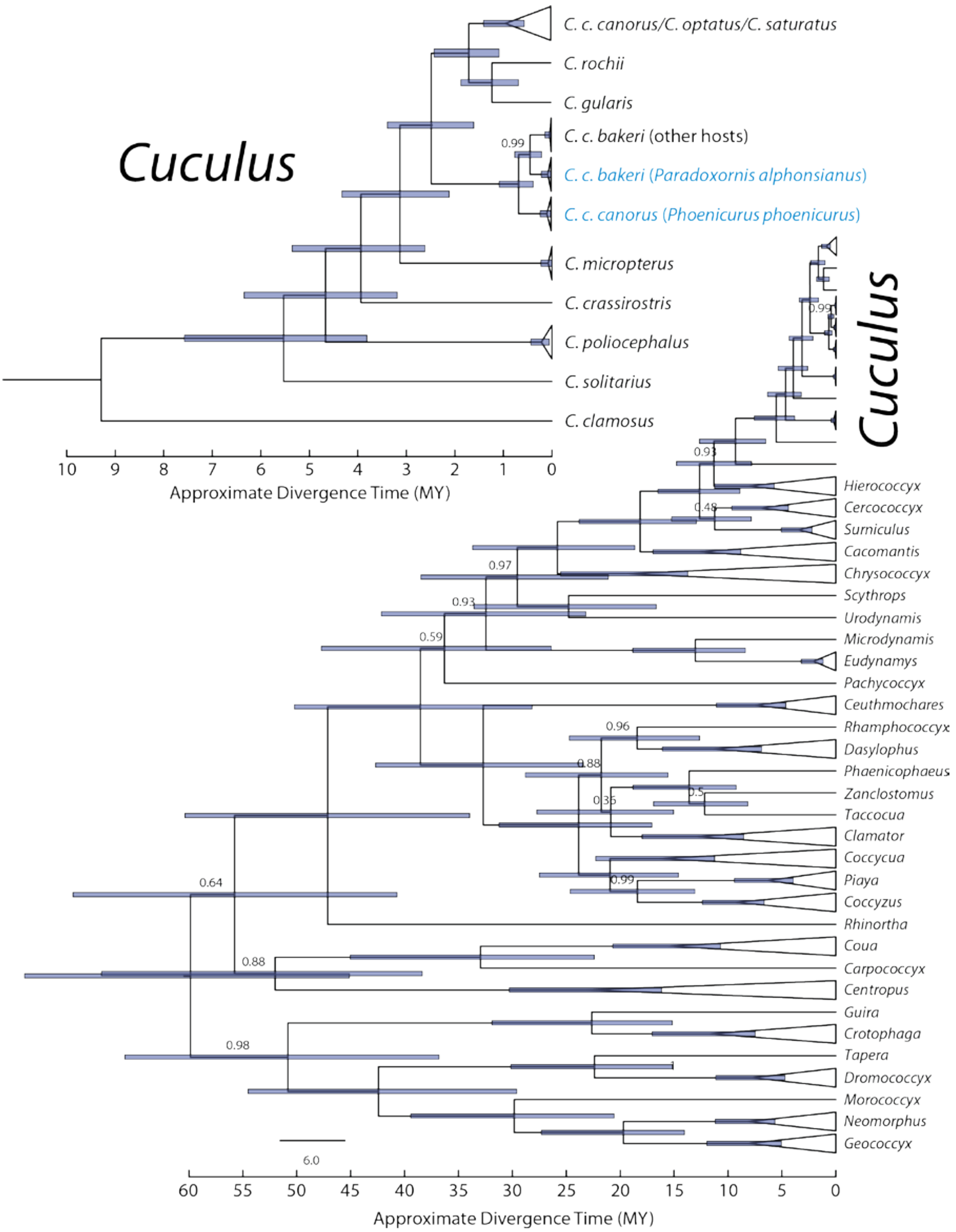

Supplementary Figure 2 | BEAST[1] analysis showing the phylogenetic relationship with approximate divergence time of Cuculiformes. Based on mitochondrial 12S and ND2 sequences representing 119 of 141 recognized cuckoo species from Sorenson and Payne (2005)[2], augmented with representative samples of additional *Cuculus* samples from the present study. Multiple samples for genera other than *Cuculus* are collapsed into a single branch. Posterior probabilities for all illustrated nodes are 1.0 unless otherwise indicated. Names of host species are listed in parentheses, and blue text denotes common cuckoos laying blue eggs (top figure). See Methods for details on the analysis.

Supplementary Table 1 | Genetic samples of European cuckoos. Samples of common cuckoo (*Cuculus canorus*) nestlings from Europe included in the population genetic analyses after assigning sibship and maternity using COLONY[3]. Among 205 genotyped cuckoo nestlings, 197 could successfully be assigned to 104 inferred mothers. *Three additional samples were added after the population genetic analyses to investigate the geographic distribution of the two divergent mitochondrial lineages (see Fig. 1).

Country	Locality	Host species		No offspring	No females	Latitude	Longitude
Bulgaria	Zlatia	Great reed warbler	*Acrocephalus arundinaceus*	33	18	43.77	23.50
Hungary	Apaj	Great reed warbler	*Acrocephalus arundinaceus*	23	13	47.12	19.10
Bulgaria	Zlatia	Marsh warbler	*Acrocephalus palustris*	23	11	43.77	23.50
Germany	Diergarten	Reed warbler	*Acrocephalus scirpaceus*	5	1	51.23	6.10
Germany	Mohrhof	Reed warbler	*Acrocephalus scirpaceus*	6	3	49.67	10.85
Czech Republic	Luzice	Reed warbler	*Acrocephalus scirpaceus*	15	6	48.83	17.07
Poland	Milicz	Reed warbler	*Acrocephalus scirpaceus*	19	13	51.53	17.37
Norway	Øvre Heimdal	Meadow pipit	*Anthus pratensis*	2	2	61.42	8.87
Norway	Hafjell	Meadow pipit	*Anthus pratensis*	1	1	61.23	10.71
Sweden	Ammarnäs	Meadow pipit	*Anthus pratensis*	1	1	65.97	16.21
Finland	Kittilä	Brambling	*Fringilla montifringilla*	4	3	67.65	24.90
Bulgaria	Zlatia	Corn bunting	*Milaria calandra*	33	15	43.77	23.50
Finland	Ruokolahti	Common redstart	*Phoenicurus phoenicurus*	32	17	61.42	28.53
			TOTAL:	197	104		
*Romania	Lake Sic	Great reed warbler	*Acrocephalus arundinaceus*	1		46.93	23.9
*Denmark	Arresø	Reed warbler	*Acrocephalus scirpaceus*	1		55.90	12.06
*Spain	Ebro delta	Reed warbler	*Acrocephalus scirpaceus*	1		40.73	0.78

Supplementary Table 2 | Pairwise genetic divergence (above diagonal) between European common cuckoo *Cuculus canorus* host races. (**a**) COI – mitochondrial DNA, (**b**) MYO – nuclear autosomal DNA (2N), (**c**) SPIN – nuclear Z chromosome DNA and (**d**) microsatellites – nuclear autosomal DNA. Genetic divergence was quantified as Φ_{ST}[11], a sequence-based homologue of F_{ST}, for the three sequenced loci. For the microsatellite loci, genetic divergence was analysed using hierarchical F_{ST}[12] and sample sizes (N) below denotes number of inferred females with number of cuckoo nestlings in parentheses. *P*-values are shown below diagonal.

a

COI	N	*P.phoenicurus*	*A.arundinaceus*	*A.scirpaceus*	*A.palustris*	*M.calandra*	*An.pratensis*	*F.montifringilla*
P.phoenicurus	17	—	0.898	0.911	0.968	0.912	0.995	0.994
A.arundinaceus	30	*<0.001*	—	~0	0.443	0.019	0.099	0.554
A.scirpaceus	20	*<0.001*	*n.s.*	—	0.501	0.051	0.008	0.574
A.palustris	11	*<0.001*	*<0.001*	*<0.001*	—	0.240	0.815	0.817
M.calandra	13	*<0.001*	*n.s.*	*n.s.*	*0.037*	—	0.261	0.502
An.pratensis	4	*0.001*	*n.s.*	*n.s.*	*0.002*	*0.099*	—	1.00
F.montifringilla	3	*0.001*	*0.001*	*0.003*	*0.005*	*0.004*	*0.030*	—

b

MYO	N	*P.phoenicurus*	*A.arundinaceus*	*A.scirpaceus*	*A.palustris*	*M.calandra*	*An.pratensis*	*F.montifringilla*
P.phoenicurus	14	—	0.010	~0	0.030	0.046	0.047	0.127
A.arundinaceus	26	*n.s.*	—	~0	0.025	~0	~0	0.040
A.scirpaceus	9	*n.s.*	*n.s.*	—	0.052	0.029	0.039	0.154
A.palustris	10	*n.s.*	*n.s.*	*n.s.*	—	0.042	~0	0.008
M.calandra	9	*0.081*	*n.s.*	*n.s.*	*n.s.*	—	~0	~0
An.pratensis	2	*n.s.*	*n.s.*	*n.s.*	*n.s.*	*n.s.*	—	~0
F.montifringilla	1	*0.076*	*n.s.*	*0.086*	*n.s.*	*n.s.*	*n.s.*	—

c

SPIN	N	*P.phoenicurus*	*A.arundinaceus*	*A.scirpaceus*	*A.palustris*	*M.calandra*	*An.pratensis*	*F.montifringilla*
P.phoenicurus	11	—	0.027	~0	~0	0.216	0.013	0.082
A.arundinaceus	17	*n.s.*	—	~0	~0	0.141	~0	~0
A.scirpaceus	4	*n.s.*	*n.s.*	—	~0	0.145	~0	~0
A.palustris	4	*n.s.*	*n.s.*	*n.s.*	—	0.148	~0	~0
M.calandra	5	*0.071*	*0.090*	*n.s.*	*n.s.*	—	~0	0.065
An.pratensis	2	*n.s.*	*n.s.*	*n.s.*	*n.s.*	*n.s.*	—	~0
F.montifringilla	2	*n.s*	*n.s.*	*n.s.*	*n.s.*	*n.s.*	*n.s.*	—

d

Microsatellites	N	*P.phoenicurus*	*A.arundinaceus*	*A.scirpaceus*	*A.palustris*	*M.calandra*	*An.pratensis*	*F.montifringilla*
P.phoenicurus	17 (32)	—	0.013	0.007	0.032	0.011	0.024	0.012
A.arundinaceus	31 (56)	*<0.001*	—	0.021	0.021	0.012	0.036	0.007
A.scirpaceus	23 (45)	*0.057*	*<0.001*	—	0.025	0.008	0.020	0.008
A.palustris	11 (23)	*<0.001*	*0.002*	*<0.001*	—	0.010	0.054	0.025
M.calandra	15 (33)	*0.018*	*<0.001*	*0.002*	*0.019*	—	0.022	~0
An.pratensis	4 (4)	*n.s.*	*0.090*	*n.s.*	*n.s.*	*n.s.*	—	0.036
F.montifringilla	3 (4)	*n.s.*	*n.s.*	*n.s.*	*n.s.*	*n.s.*	*n.s.*	—

Supplementary Table 3 | Genetic samples of Asian and African cuckoos. Samples of cuckoo species from Asia and Africa included in the phylogenetic analyses.

Country	Locality	Cuckoo species		Host species		N	Latitude	Longitude
China	Guizhou	Common cuckoo	*Cuculus c. bakeri*	Ashy-throated parrotbill	*Paradoxornis alphonsianus*	5	28.2	107.2
China	Guizhou	Common cuckoo	*Cuculus c. bakeri*	White wagtail	*Motacilla alba*	1	28.2	107.2
China	Shandong	Common cuckoo	*Cuculus c. bakeri*	Oriental reed warbler	*Acrocephalus orientalis*	3	37.7	118.9
China	Shanghai	Common cuckoo	*Cuculus c. bakeri*	Oriental reed warbler	*Acrocephalus orientalis*	3	31.4	121.8
China	Heilongjiang	Common cuckoo	*Cuculus c. bakeri*	Oriental reed warbler	*Acrocephalus orientalis*	2	46.8	123.9
China	Gansu	Common cuckoo	*Cuculus c. canorus*	Rufous-tailed shrike	*Lanius isabellinus*	2	40.3	96.1
Zambia	Choma	African cuckoo	*Cuculus gularis*			4	-16.8	26.9
Bangladesh	Dhaka	Indian cuckoo	*Cuculus micropterus*	Black drongo	*Dicrurus macrocercus*	2	23.9	90.3
China	Henan	Indian cuckoo	*Cuculus micropterus*	Black drongo	*Dicrurus macrocercus*	4	31.7	114.3
Singapore		Indian cuckoo	*Cuculus micropterus*			1	1.32	103.8
China	Guizhou	Lesser cuckoo	*Cuculus poliocephalus*	Brownish-flanked bush-warbler	*Cettia fortipes*	1	28.2	107.2
Madagascar	Sakaraha	Madagascar cuckoo	*Cuculus rochii*			1	-22.7	44.8
China	Guizhou	Himalayan cuckoo	*Cuculus saturatus*	Yellow-throated bunting	*Emberiza elegans*	1	28.2	107.2
China	Guizhou	Himalayan cuckoo	*Cuculus saturatus*	Yellowish-bellied bush-warbler	*Cettia acanthizoides*	1	28.2	107.2
China	Guizhou	Large hawk- cuckoo	*Hierococcyx sparverioides*	Chinese babax	*Babax lanceolatus*	1	28.2	107.2
China	Guizhou	Large hawk- cuckoo	*Hierococcyx sparverioides*	White-browed laughingthrush	*Garrulax sannio*	1	28.2	107.2
Bangladesh	Dhaka	Common hawk-cuckoo	*Hierococcyx varius*	Jungle babbler	*Turdoides striatus*	2	23.9	90.3

Supplementary Table 4 | Genbank accession numbers. Additional COI sequences downloaded from Genbank.

Genbank	Voucher	Cuckoo species		Country	Locality
AB842683	BJNSM381-10	Common cuckoo	*Cuculus canorus*	Japan	Nagano
AB842684	BJNSM378-10	Common cuckoo	*Cuculus canorus*	Japan	Nagano
AB842685	BJNSM379-10	Common cuckoo	*Cuculus canorus*	Japan	Nagano
EF515762	KRIBB2279	Common cuckoo	*Cuculus canorus*	South Korea	South Cholla
GQ481655	UWBM 56594	Common cuckoo	*Cuculus canorus*	Russia	Astrakhan'
GQ481656	UWBM 47024	Common cuckoo	*Cuculus canorus*	Russia	Stekol'nyy
GQ481657	UWBM 66441	Common cuckoo	*Cuculus canorus*	Russia	Kyzyl
GQ481658	UWBM 58010	Common cuckoo	*Cuculus canorus*	Mongolia	Hangayn Nuruu
GQ481659	UWBM 46365	Common cuckoo	*Cuculus canorus*	Kazakhstan	Akkol'
GU571356	NHMO-BC47	Common cuckoo	*Cuculus canorus*	Norway	Utsira
GU571357	NHMO-BC304	Common cuckoo	*Cuculus canorus*	Norway	Jomfruland
GU571847	BISE-Aves292	Common cuckoo	*Cuculus canorus*	Sweden	Södertälje
GU571848	BISE-Aves143	Common cuckoo	*Cuculus canorus*	Sweden	Åkersberga
JN801307	USNM 621533	Common cuckoo	*Cuculus canorus*	Mongolia	Hangayn Nuruu
AB842691	BJNSM231-10	Lesser cuckoo	*Cuculus poliocephalus*	Japan	Nagano
AB842692	BJNSM614-10	Lesser cuckoo	*Cuculus poliocephalus*	Japan	Sakishima Isls.
AB842693	BJNSM604-10	Lesser cuckoo	*Cuculus poliocephalus*	Japan	Amami Isls.
AB842688	BJNSM135-10	Oriental cuckoo	*Cuculus optatus*	Japan	Hyogo
AB842689	BJNSM218-10	Oriental cuckoo	*Cuculus optatus*	Japan	Obihiro-shi
AB842690	BJNSM683-10	Oriental cuckoo	*Cuculus optatus*	Japan	Sakishima Isls.
GQ481660	UWBM 44563	Oriental cuckoo	*Cuculus optatus*	Russia	Koryaki
GQ481661	UWBM 44401	Oriental cuckoo	*Cuculus optatus*	Russia	Magadan
GQ481662	UWBM 56743	Oriental cuckoo	*Cuculus optatus*	Russia	Noyabr'sk
GQ481663	UWBM 71703	Oriental cuckoo	*Cuculus optatus*	Russia	Rudnaya Pristan'
GQ481664	UWBM 49457	Oriental cuckoo	*Cuculus optatus*	Russia	Nizhniye Sergi
AB842686	BJNSM436-10	Rufous hawk-cuckoo	*Hierococcyx hyperythrus*	Japan	Tokachi-shicho
AB842687	BJNSM498-10	Rufous hawk-cuckoo	*Hierococcyx hyperythrus*	Japan	Tokachi-shicho

Supplementary Table 5 | Pairwise genetic divergence (above diagonal) between selected lineages of the common cuckoo *Cuculus canorus*. (**a**) COI – mitochondrial DNA, (**b**) MYO – nuclear autosomal DNA (2N) and (**c**) SPIN – nuclear Z chromosome DNA. Genetic divergence was quantified as Φ_{ST}[11], a sequence-based homologue of F_{ST}. *P*-values are shown below diagonal.

a

COI	N	*C. c. canorus (P. phoenicurus)*	*all other C. c. canorus*	*C. c. bakeri*
C. c. canorus/P. phoenicurus	17	—	0.861	0.596
all other C. c. canorus	78	*<0.001*	—	0.740
C. c. bakeri	12	*<0.001*	*<0.001*	—

b

MYO	N	*C. c. canorus (P. phoenicurus)*	*all other C. c. canorus*	*C. c. bakeri*
C. c. canorus/P. phoenicurus	18 (36)	—	0.019	~0
all other C. c. canorus	69 (138)	*n.s.*	—	0.005
C. c. bakeri	9 (18)	*n.s.*	*n.s.*	—

c

SPIN	N	*C. c. canorus (P. phoenicurus)*	*all other C. c. canorus*	*C. c. bakeri*
C. c. canorus/P. phoenicurus	11	—	0.087	0.226
all other C. c. canorus	34	*0.092*	—	~0
C. c. bakeri	6	*n.s.*	*n.s.*	—

Supplementary Table 6 | Genetic samples of museum eggshells. Samples of common cuckoo *Cuculus canorus* museum eggs genotyped for the short COI sequence from V. Čapek's collection stored in the Moravian Museum, Budisov, Czech Republic. Mitochondrial lineages corresponded perfectly with egg colouration (immaculate blue versus speckled egg types), irrespective of host species. The speckled cuckoo eggs found in redstart nests and blue cuckoo eggs found in the nests of hosts with speckled eggs were therefore likely 'mis-layings' by females belonging to the other mitochondrial lineage. Some cuckoo females are known to lay their eggs in nests of more than one host species[4,5]

Museum ID	Year	Locality	Host species		Egg type	Mitochondrial DNA lineage
MZM4888	1896	NA	Meadow pipit	*Anthus trivialis*	**Blue**	***bakeri***
MZM4429	1901	Budkovice	European robin	*Erithacus rubecula*	**Blue**	***bakeri***
MZM4524	1907	Chvojnice	European robin	*Erithacus rubecula*	**Blue**	***bakeri***
MZM4830	1904	Zbejsov	European robin	*Erithacus rubecula*	**Blue**	***bakeri***
MZM4862	1897	Neslovice	Red-backed shrike	*Lanius collurio*	**Blue**	***bakeri***
MZM4899	1909	Padochov	White wagtail	*Motacilla alba*	**Pale blue**	***bakeri***
MZM4900	1898	Nova Ves	White wagtail	*Motacilla alba*	**Blue**	***bakeri***
MZM4739	1894	Padochov	Common redstart	*Phoenicurus phoenicurus*	**Pale blue**	***bakeri***
MZM4746	1902	Nova Ves	Common redstart	*Phoenicurus phoenicurus*	**Blue**	***bakeri***
MZM4748	1907	Nova Ves	Common redstart	*Phoenicurus phoenicurus*	**Blue**	***bakeri***
MZM4753	1906	Padochov	Common redstart	*Phoenicurus phoenicurus*	**Blue**	***bakeri***
MZM4753	1906	Padochov	Common redstart	*Phoenicurus phoenicurus*	**Speckled**	***canorus***
MZM4755	1910	Ivancice	Common redstart	*Phoenicurus phoenicurus*	**Blue**	***bakeri***
MZM4756	1899	Ivancice	Common redstart	*Phoenicurus phoenicurus*	**Blue**	***bakeri***
MZM4758	1912	Ivancice	Common redstart	*Phoenicurus phoenicurus*	**Blue**	***bakeri***
MZM4759	1901	Rena	Common redstart	*Phoenicurus phoenicurus*	**Blue**	***bakeri***
MZM4762	1902	Budkovice	Common redstart	*Phoenicurus phoenicurus*	**Blue**	***bakeri***
MZM4763	1908	Budkovice	Common redstart	*Phoenicurus phoenicurus*	**Blue**	***bakeri***
MZM4764	1909	Budkovice	Common redstart	*Phoenicurus phoenicurus*	**Blue**	***bakeri***
MZM4765	1907	Budkovice	Common redstart	*Phoenicurus phoenicurus*	**Pale blue**	***bakeri***
MZM4766	1908	Budkovice	Common redstart	*Phoenicurus phoenicurus*	**Pale blue**	***bakeri***
MZM4769	1911	Budkovice	Common redstart	*Phoenicurus phoenicurus*	**Pale blue**	***bakeri***
MZM4769	1911	Budkovice	Common redstart	*Phoenicurus phoenicurus*	**Blue**	***bakeri***
MZM4775	1906	Oslavany	Common redstart	*Phoenicurus phoenicurus*	**Speckled**	***canorus***
MZM4777	1892	Oslavany	Common redstart	*Phoenicurus phoenicurus*	**Speckled**	***canorus***
MZM4782	1909	Oslavany	Common redstart	*Phoenicurus phoenicurus*	**Speckled**	***canorus***
MZM4783	1905	Oslavany	Common redstart	*Phoenicurus phoenicurus*	**Speckled**	***canorus***
MZM4785	1908	Zbejsov	Common redstart	*Phoenicurus phoenicurus*	**Speckled**	***canorus***
MZM4788	1900	Ivancice	Common redstart	*Phoenicurus phoenicurus*	**Speckled**	***canorus***
MZM4789	1907	Hliny	Common redstart	*Phoenicurus phoenicurus*	**Speckled**	***canorus***
MZM4791	1897	Rena	Common redstart	*Phoenicurus phoenicurus*	**Speckled**	***canorus***
MZM4334	1891	Oslavany	Wood warbler	*Phylloscopus sibilatrix*	**Blue**	***bakeri***
MZM4359	1909	Budkovice	Garden warbler	*Sylvia borin*	**Blue**	***bakeri***

Supplementary Table 7 | Genetic samples of blue museum eggshells. Blue common cuckoo *Cuculus canorus* eggs from museums genotyped for the short COI sequence from the Natural History Museum, University of Oslo, Norway (NHMO) and Natural History Museum, Tring, UK (NHMUK). All eggs belonged to the *bakeri* mitochondrial lineage.

Museum ID	Year	Country	Locality	Host species	
NHMO-LE3198	1892	Norway	Hvalstad	Winchat	*Saxicola rubetra*
NHMUK-1873	1873	Greece	Parnassus	Blackeared wheatear	*Oenanthe hispanica*
NHMUK-85G1	1880	Netherlands	Valkenwaard	Common redstart	*Phoenicurus phoenicurus*
NHMUK-1919.21.5289	1899	Hungary	Csepel Island	Common redstart	*Phoenicurus phoenicurus*
NHMUK-Baker Box48/D2	1907	Pakistan	Thandiani	*(oviduct fragment, unknown host race)*	
NHMUK-1925.12.15.5852	1914	Pakistan	Murree	Rufous-bellied niltava	*Niltava sundara*
NHMUK-1996.1.126	1923	Burma	NA	Pied bushchat	*Saxicola caprata*

Supplementary Table 8 | Genetic markers. PCR primers and characteristics of loci used for DNA sequencing. The three new primer pairs designed in this study were used to sequence the African cuckoo *Cuculus gularis* and Madagacar cuckoo *C. rochii* (Spindlin), the common cuckoo *C. canorus* (CHDW) and the museum eggshell samples (COI).

Locus	Primer	Target	Size (bp)	Primer sequence (5' - 3')
Spindlin[6]	Spin319F	ChrZ, Intron2	951	TATGGACTAGAACTGCACAAAG
	Spin472R			AGACCATCCCCCTCCATTCATC
Spindlin[This study]	SpinCuF	ChrZ, Intron2	856	CAGCACTTGAAGTTCTTCCAGAC
	SpinCuR			GCTTTACCAATCATTGTGTCTGCC
Myoglobin[6]	MYO2	Chr1, Intron2	721	GCCACCAAGCACAAGATCCC
	MYO3F			TTCAGCAAGGACCTTGATAATGACTT
COI[7]	Bird F1	mtDNA	695	TTCTCCAACCACAAAGACATTGGCAC
	Bird R1			ACGTGGGAGATAATTCCAAATCCTG
COI[This study]	COI-CuF	mtDNA	159	CATCACAACAGCCATCAACA
	COI-CuR			TAATACGGGGTCACCTCCTC
ND2[2]	L5216	mtDNA	1087	GGCCCATACCCCGRAAATG
	H5766			RGAKGAGAARGCYAGGATYTTKCG
	L5758			GGNGGNTGAATRGGNYTNAAYCARAC
	H6313			ACTCTTRTTTAAGGCTTTGAAGGC
12S[2]	L1263	mtDNA	1027	YAAAGCATGRCACTGAA
	H1859			TCGDTTRYAGRACAGGCTCCTCTA
	L1754			TGGGATTAGATACCCCACTATG
	H2294			TYTCAGGYGTARGCTGARTGCTT
CHDW[This study]	CHDwF	ChrW	443	CATTCAGATCACCGTTTCACTTC
	CHDwR			GTAAACATTTTCCATTTCTTGGAGG

Supplementary Table 9 | Microsatellite markers. Summary statistics for microsatellite loci genotyped in European common cuckoos *Cuculus canorus*. Observed (H_O) and expected (H_E) heterozygosity is shown with *P*-values for tests of deviations from Hardy-Weinberg equilibrium for each marker.

Locus	N	No alleles	Allele size range	H_O	H_E	*P*
Ccy1[8]	104	4	6	0.25	0.26	0.36
Ccy9[8]	104	4	4	0.54	0.51	0.63
Ccy13[8]	104	8	18	0.77	0.74	0.52
Ccy60[9]	104	26	51	0.92	0.90	0.36
Ccy88[9]	104	11	20	0.78	0.79	0.27
Ccy100[9]	101	6	12	0.65	0.72	0.33
Ccy108[9]	104	9	22	0.63	0.61	0.40
Ccy119[9]	104	32	48	0.88	0.94	0.018
Ccy137[9]	104	23	48	0.77	0.91	0.001
Cba8[10]	104	31	47	0.90	0.94	0.008
Clu2[10]	99	8	30	0.64	0.67	0.26
Clu3[10]	104	5	4	0.38	0.42	0.59
Clu5[10]	103	16	24	0.90	0.88	0.17

Supplementary Table 10 | Estimates of divergence time for the basal split between the New World cuckoos, comprising Crotophaginae and Neomorphinae, and the predominantly Old World clade comprising all other cuckoos.

Age	Reference
69.0 MY	Sibley & Ahlquist 1990[13]
52.0 MY	van Tuinen & Hedges 2001[14]
~47-52 MY	Ericson et al. 2006[15]
~54.4 MY	Brown et al. 2007[16]
52.5-79.5 MY	Brown et al. 2008[17]
59.76-63.38 MY	Pacheco et al. 2011[18]

Supplementary References

1. Drummond, A. J. *et al.* Bayesian phylogenetics with BEAUti and the BEAST 1.7. *Mol. Biol. Evol.* **29**, 1969-1973 (2012).

2. Sorenson, M. D. & Payne, R. B. in *The cuckoos* (eds R. B. Payne, M. D. Sorenson, K. Klitz, & J. Megahan) 68-94 (Oxford University Press, 2005).

3. Jones, O. R. & Wang, J. COLONY: A program for parentage and sibship inference from multilocus genotype data. *Mol. Ecol. Resour.* **10**, 551-555 (2010).

4. Fossøy, F. *et al.* Genetic differentiation among sympatric cuckoo host races: males matter. *Proc. R. Soc. B* **278**, 1639-1645 (2011).

5. Marchetti, K., Nakamura, H. & Gibbs, H. L. Host-race formation in the common cuckoo. *Science* **282**, 471-472 (1998).

6. Kimball, R. T. *et al.* A well-tested set of primers to amplify regions spread across the avian genome. *Mol. Phylogenet. Evol.* **50**, 654-660 (2009).

7. Hebert, P. D. N. *et al.* Identification of birds through DNA barcodes. *PLoS Biol.* **2**, 1657-1663 (2004).

8. Gibbs, H. L., Brooke, M. D. & Davies, N. B. Analysis of genetic differentiation of host races of the common cuckoo *Cuculus canorus* using mitochondrial and microsatellite DNA variation. *Proc. R. Soc. B* **263**, 89-96 (1996).

9. Gibbs, H. L. *et al.* Isolation and characterization of microsatellite DNA loci for an obligate brood parasitic bird, the common cuckoo (*Cuculus canorus*). *Mol. Ecol.* **7**, 1437-1439 (1998).

10. Adcock, G. J., Langmore, N. E. & Kilner, R. M. Polymorphic microsatellite loci for studies of bronze-cuckoo species (Genus Chalcites: Aves). *Mol. Ecol. Notes* **7**, 678-680 (2007).

11. Excoffier, L., Smouse, P. E. & Quattro, J. M. Analysis of molecular variance inferred from metric distances among DNA haplotypes - application to human mitochondrial DNA restriction data. *Genetics* **131**, 479-491 (1992).

12. Goudet, J. Hierfstat, a package for R to compute and test hierarchical *F*-statistics. *Mol. Ecol. Notes* **5**, 184-186 (2005).

13. Sibley, C. & Ahlquist, J. *Phylogeny and classification of birds: A study in molecular evolution*. (Yale University Press, 1990).

14. van Tuinen, M. & Hedges, S. B. Calibration of avian molecular clocks. *Mol. Biol. Evol.* **18**, 206-213 (2001).

15. Ericson, P. G. P. *et al.* Diversification of Neoaves: Integration of molecular sequence data and fossils. *Biol. Letters* **2**, 543-547 (2006).

16. Brown, J. W., Payne, R. B. & Mindell, D. P. Nuclear DNA does not reconcile 'rocks' and 'clocks' in Neoaves: a comment on Ericson et al. *Biol. Letters* **3**, 257-260 (2007).

17. Brown, J. W. *et al.* Strong mitochondrial DNA support for a Cretaceous origin of modern avian lineages. *BMC Biology* **6**, 1-18 (2008).

18. Pacheco, M. A. *et al.* Evolution of modern birds revealed by mitogenomics: Timing the radiation and origin of major orders. *Mol. Biol. Evol.* **28**, 1927-1942 (2011).
